# Breaking the Limit of Cardiovascular Regenerative Medicine: Successful 6-Month Goat Implant in World’s First Ascending Aortic Replacement Using Biotube Blood Vessels

**DOI:** 10.3390/bioengineering11040405

**Published:** 2024-04-20

**Authors:** Kazuki Mori, Tadashi Umeno, Takayuki Kawashima, Tomoyuki Wada, Takuro Genda, Masanagi Arakura, Yoshifumi Oda, Takayuki Mizoguchi, Ryosuke Iwai, Tsutomu Tajikawa, Yasuhide Nakayama, Shinji Miyamoto

**Affiliations:** 1 Department of Cardiovascular Surgery, Oita University, Oita 879-5593, Japan; umenot@oita-u.ac.jp (T.U.); t-kawashima@oita-u.ac.jp (T.K.); wada@oita-u.ac.jp (T.W.); smiyamot@oita-u.ac.jp (S.M.); 2 Department of Clinical Engineering, Oita University Hospital, Oita 879-5593, Japant-mizoguchi@oita-u.ac.jp (T.M.); 3 Institute of Frontier Science and Technology, Okayama University of Science, Okayama 700-0005, Japan; iwai@ous.ac.jp; 4 Department of Mechanical Engineering, Faculty of Engineering Science, Kansai University, Osaka 564-8680, Japan; tajikawa@kansai-u.ac.jp; 5 Osaka Laboratory, Biotube Co., Ltd., Osaka 565-0842, Japan; y.nakayama@biotube.co.jp

**Keywords:** in-body tissue architecture technology, tissue engineering, Biotube, vascular prosthesis, regenerative medicine, cardiovascular surgery

## Abstract

This study investigated six-month outcomes of first models of ascending aortic replacement. The molds used to produce the Biotube were implanted subcutaneously in goats. After 2–3 months, the molds were explanted to obtain the Biotubes (inner diameter, 12 mm; wall thickness, 1.5 mm). Next, we performed ascending aortic replacement using the Biotube in five allogenic goats. At 6 months, the animals underwent computed tomography (CT) and histologic evaluation. As a comparison, we performed similar surgeries using glutaraldehyde-fixed autologous pericardial rolls or pig-derived heterogenous Biotubes. At 6 months, CT revealed no aneurysmalization of the Biotube or pseudoaneurysm formation. The histologic evaluation showed development of endothelial cells, smooth muscle cells, and elastic fibers along the Biotube. In the autologous pericardium group, there was no evidence of new cell development, but there was calcification. The histologic changes observed in the heterologous Biotube group were similar to those in the allogenic Biotube group. However, there was inflammatory cell infiltration in some heterologous Biotubes. Based on the above, we could successfully create the world’s first Biotube-based ascending aortic replacement models. The present results indicate that the Biotube may serve as a scaffold for aortic tissue regeneration.

## 1. Introduction

A vascular prosthesis made of polyester fiber is used in aortic surgery worldwide. Given the stable durability and clinical results of these grafts, they are commonly used for aortic replacement. However, once a vascular prosthesis becomes infected, it can cause serious problems such as sepsis [1,2]. In contrast, grafts made of biological materials have the potential to prevent infectious complications. Despite previous efforts aiming to develop tissue-engineered grafts [3], the use of these materials has not yet been clinically tested.

In-body tissue architecture (iBTA) technology is a technology for the production of biogenic grafts based on the tissue encapsulation of foreign material. iBTA technology can produce autologous tissue grafts by subcutaneously implanting the mold into the body. Biotubes are formed by collagen fibers produced by fibroblasts invading the gap between a cylindrical outer tube with multi-holes and a core rod. The graft shape and size depend on the mold. At our institution, we have used iBTA technology to create aortic valve replacement material and peripheral vascular grafts and reported our results in animal models [4,5,6,7]. iBTA technology has potential applications in aortic surgery. This study aimed to evaluate the in vivo performance of aortic Biotubes, which are tubular grafts made using iBTA technology. An autologous pericardium was used as a biogenous graft for comparison. In addition, Biotube formation was observed ~1 month after mold implantation under the skin and required >2 months to achieve sufficient collagen fiber density [5,7]. We do not expect that Biotubes are indicated in urgent surgery due to their unfavorable formation time. Therefore, we also experimented with transplantation of a heterologous Biotube.

## 2. Materials and Methods

### 2.1. Biotube Production

**Figure 1:**
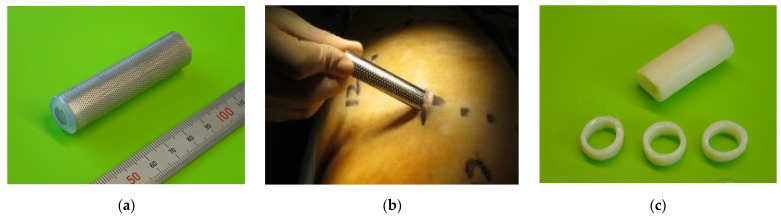

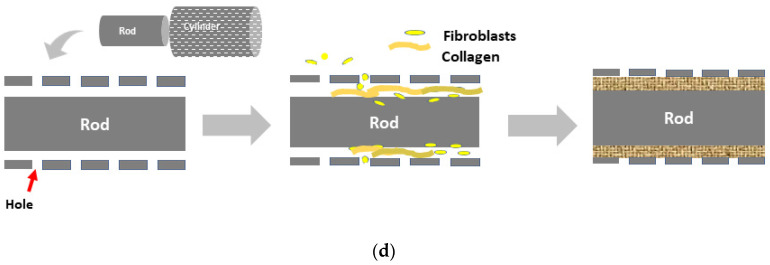
(**a**) Mold appearance. (**b**) Molds were implanted subcutaneously in animals for 2–3 months. (**c**) Biotube after removal from the mold. (**d**) Fibroblasts penetrating the gap between the outer cylinder and inner rod as shown in the mold schema. The tissue-engineered graft is formed by collagen according to gap shape.

The molds were assembled by inserting a silicone tube (outer diameter, 12 mm; length, 60 mm) with an acryl rod into a stainless-steel pipe (outer diameter, 15 mm; length, 60 mm) with multiple small round holes (Figure 1a). Both edges were capped to ensure that the silicone tube was centered. The gap between the tube and pipe was 1.0 mm. Fibroblasts will penetrate the gap between the outer cylinder and inner rod, as shown in Figure 1c. This tissue-engineered graft is formed by collagen filling in the gap. Next, the molds were embedded in the abdominal subcutaneous pouch of adult Saanen goats (≥12 months; weight, 53.7 ± 6.1 kg) under general anesthesia (induced with 2 mg/kg of ketamine and maintained with 2–3% sevoflurane; Figure 1b). After 2–3 months, the molds were explanted, and the Biotube formed within was extracted. Then, the Biotube was dehydrated with 70% ethanol to increase the collagen density. This process resulted in a Biotube with an inner diameter of 12 mm, a wall thickness of 1.5 mm, and a length of 60 mm (Figure 1c). The Biotube was kept in ethanol until the aortic surgery. Heterogeneous Biotubes were prepared using the same procedure in adult pigs and decellularized to avoid immune reactions.

### 2.2. Aortic Replacement Procedure

**Figure 2:**
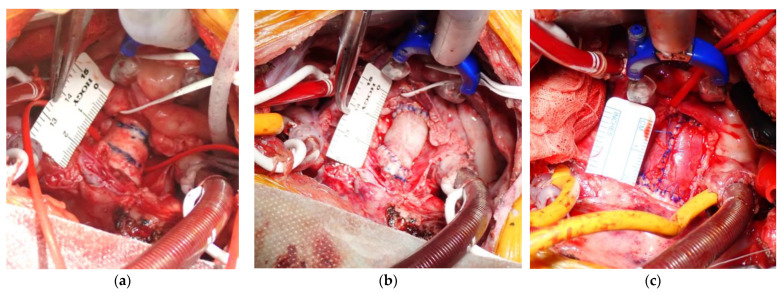
Intraoperative images of aortic replacement. Prereplacement picture (**a**) with Biotube (**b**) and autologous pericardial roll (**c**). The aorta was resected between the blue lines shown in (**a**). The length of replacement was approximately 20 mm.

Young Saanen goats (6–7 months old; weight, 27.1 ± 3.6 kg) underwent left thoracotomy under general anesthesia. The anesthesia was induced with 2 mg/kg ketamine and maintained with 2–3% sevoflurane. The heart was exposed by incising the pericardium. A cardiopulmonary bypass was established by cannulating the distal aortic arch and right atrium after administering heparin (400 U/kg). After aortic cross-clamping, the heart was arrested by antegrade cold crystalloid cardioplegia. Then, the ascending aorta was exposed by approximately 2.0 cm. After rinsing the Biotube in 70% ethanol with saline, the Biotube was anastomosed to the aorta through continuous sutures on the posterior wall and nodal sutures on the anterior wall using 5-0 monofilament sutures (Figure 2a,b). When the animal was weaned from cardiopulmonary bypass, the wound was closed after ensuring lack of bleeding in the thoracic cavity.

As controls, some goats underwent aortic replacement using heterogeneous Biotubes or autologous pericardial rolls. In the pericardial roll group, the pericardium was harvested after the left thoracotomy and treated with a 0.6% glutaraldehyde solution for 10 min. Then, the treated pericardium was rinsed thrice with saline for 6 min, and a 12 mm diameter pericardial roll was formed using a 5 cc syringe as a core with 5-0 monofilament nodal sutures (Figure 2c). The aortic replacement procedure in both control groups was as described above for the allogenic Biotube group.

In all groups, no medication was required after postoperative recovery. Contrast-enhanced computed tomography (CT) was performed under general anesthesia 6 months postoperatively. The heart and aorta were then exposed by performing a left thoracotomy along the previous wound. After exposing the aorta, a direct aortic echography was performed to assess the shape and lumen of the graft. After collecting data, heparin (100 U/kg) was administered, and the animal was euthanized by injection of pentobarbital sodium (120 mg/kg) and KCL (2 mEq/kg). Finally, the heart and aorta, including the replacement site, were extracted.

### 2.3. Imaging and Histologic Tests

We examined the presence of tears or perforations, aneurysms, thrombosis, and visible calcification in the harvested Biotube. Next, the harvested tissues were fixed in a 4% paraformaldehyde saline solution (FUJIFILM Wako Pure Chemical Co., Osaka, Japan). These specimens were cut into strips every 1 cm, including the anastomosis, embedded in paraffin, sliced into 5 μm sections, and stained with hematoxylin–eosin (HE), Masson’s trichrome, Elastica van Gieson, and von Kossa at the Institute of Frontier Science and Technology, Okayama University of Science. Further, immunohistochemical staining was performed using anti-α-smooth muscle actin (α-SMA) mouse monoclonal antibodies (1:200; 904601, BioLegend, San Diego, CA, USA). Alexa Fluor^®^ 594 donkey anti-mouse secondary antibodies (1:1000; ab150108, Abcam, Cambridge, UK) were used to assess myofibroblast localization. CD31 rabbit polyclonal antibodies (1:50; ab28364, Abcam) and goat secondary antibodies to rabbit immunoglobulin G (Alexa Fluor^®^ 488) were used to confirm vascular endothelial cell localization. DAPI (ProLongTM Gold Antifade Mountant with DAPI, Thermo Fisher Scientific, Inc., Waltham, MA, USA) was used as a nuclear counterstain.

### 2.4. Statistical Analysis

Data are presented as mean ± standard deviation.

### 2.5. Ethical Approval

The animals were cared for in accordance with the Guide for the Care and Use of Laboratory Animals published by the US National Institutes of Health (NIH Publication No. 85-23, revised 1996). The Oita University Animal Ethics Committee approved all animal studies (Protocol No. 232201).

## 3. Results

### 3.1. Procedural Results

A total of 14 molds were implanted in three adult goats; Biotubes were successfully obtained from all molds after 2–3 months. The Biotubes were inspected for holes and damage and stored in 10% ethanol after a material durability test. The tensile test confirmed 22.18 ± 6.26 N. This showed that the Biotubes were strong enough to withstand >5 N, the standard strength for vascular prosthesis.

Six young goats in the allogenic Biotube group, three goats in the heterogeneous Biotube group, and three goats in the autologous pericardium group survived six months without health issues. In the allogenic Biotube group, the operating time was 251.8 ± 36.1 min, the cardiopulmonary bypass time was 105.7 ± 14.9 min, and the aortic clamping time was 64.2 ± 11.9 min. In the heterogeneous Biotube group, the operating time was 292.0 ± 39.6 min, the cardiopulmonary bypass time was 145.5 ± 20.5 min, and the aortic clamping time was 84.5 ± 12.0 min. In the autologous pericardial group, the operating time was 271.0 ± 7.0 min, the cardiopulmonary bypass time was 107.0 ± 11.5 min, and the aortic clamping time was 62.3 ± 11.4 min. No animal experienced an aortic event, including aortic rupture, during the observation period.

### 3.2. Image Evaluation

**Figure 3:**
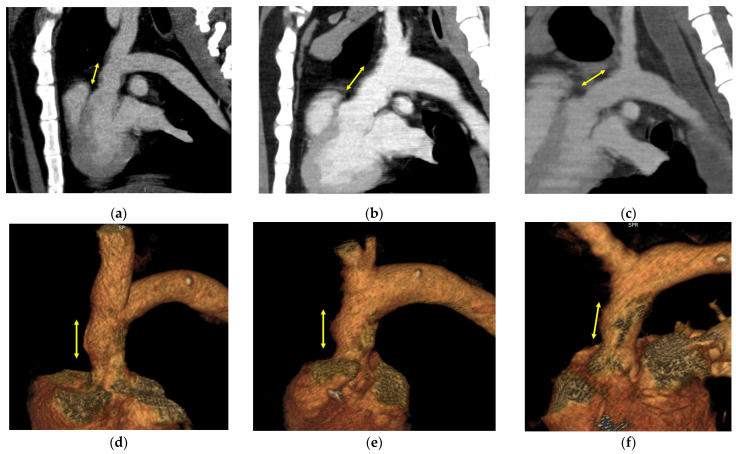
Contrast-enhanced CT image at 6 months postoperatively. Allogenic Biotube (**a**,**d**), heterologous Biotube (**b**,**e**), and autologous pericardial graft (**c**,**f**). The section indicated by the yellow arrow is the replacement area. There was no evidence of calcification, aneurysmalization, rupture, or pseudoaneurysm in any group.

Contrast-enhanced CT and echography were performed before tissue harvesting. No animals showed formation of a graft aneurysm or pseudoaneurysm. The graft diameter was 18.6 ± 2.6 mm as measured by CT in the allogenic Biotube group. In the heterogeneous Biotube group, the diameter was 20.7 ± 2.3 mm, and in the autologous pericardium group, it was 18.7 ± 2.7 mm. Figure 3 shows the CT images in each group.

**Figure 4:**
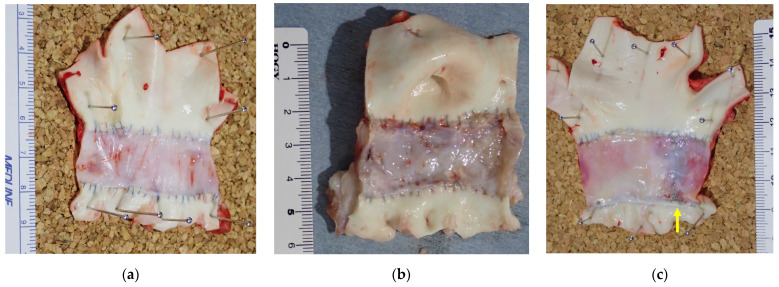
Biotubes harvested after 6 months. In the allogenic Biotube group (**a**), the luminal surface of the graft was smooth, and there was no evidence of thrombi. In the heterogeneous Biotube group (**b**), there was ulcer-like damage over the whole luminal surface but no evidence of thrombus. In the autologous pericardium group (**c**), most of the luminal surface of the graft was smooth, but the luminal surface of the roll’s suture line (yellow arrow) was rough. However, there was no evidence of thrombus.

Figure 4 shows the macroscopic findings of harvested grafts in each group. The Biotubes showed a smooth luminal surface similar to a native aorta without tears, atrophy, hypertrophy, or calcification in the allogenic Biotube group. Further, there was no evidence of graft damage or rupture in either the heterogeneous Biotube or autologous pericardium groups. However, ulcer-like lesions were observed on the surface of the Biotube lumen in the heterogeneous Biotube group. In addition, in the autologous pericardium group, the surface around the suture line of the pericardial roll was rough and calcified. In contrast, the remaining pericardial surface was smooth and uncalcified.

### 3.3. Histologic Evaluation

**Figure 5:**
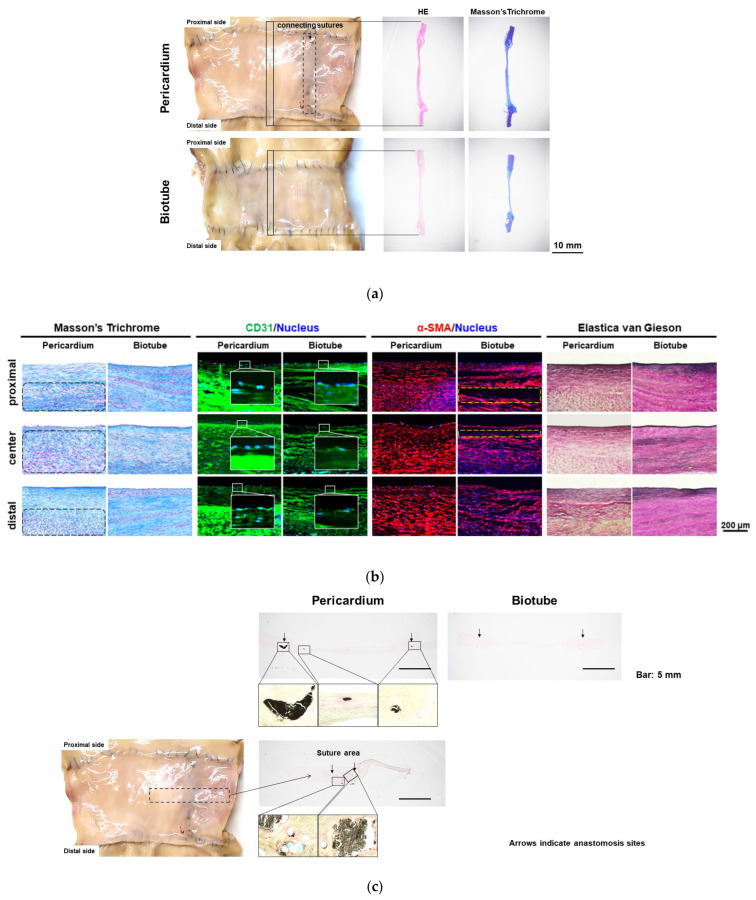
Histologic findings in the allogenic Biotube and autologous pericardium groups. (**a**) HE-stained images showing the neoplastic cell layer on the luminal side in both groups. No inflammatory cells were observed around the graft. (**b**) Masson’s trichrome staining showed the Biotube layer formed by collagen was preserved. Cells on the luminal surface were stained with CD31 immunostaining in both groups. The neoplastic cell layer was stained with α-SMA immunostaining in both groups. The Biotube layer is surrounded by yellow dashed lines. Elastica van Gieson staining showed the development of elastic fibers in the neoplastic cell layer. The Biotube group had a higher elastic fiber density. (**c**) von Kossa staining showed calcification around the suture lines in the autologous pericardium group. No calcification was observed in the Biotube group.

Figure 5 shows the histologic findings of grafts harvested at 6 months in the allogenic Biotube and autologous pericardium groups. No inflammatory cells were observed. Masson’s trichrome staining revealed that the collagen layer of the Biotube was preserved. No pericardial degradation was observed in the autologous pericardium group. There were layers of neoplastic cells along both the internal and external surfaces of the Biotubes. Similar results were observed in the autologous pericardium group. Further, cellular neoplasia was observed between the collagen fibers in the Biotube group.

Immunostaining for CD31 showed the presence of endothelial cells on the luminal side along the entire Biotube and pericardium. Furthermore, immunostaining revealed that α-SMA-positive cells developed in the neoplasia cell layer of cells in both groups. There was greater development of neoplasia cell layers in the vicinity of the anastomosis. Elastica van Gieson staining showed the development of elastic fibers in the neoplastic cell layer. Elastic fiber development was also observed in the autologous pericardium group. Elastic fiber density was higher in the Biotube group. In addition, elastic fibers developed on the outside in the Biotube group. Calcification was present around the anastomosis and suture line in the autologous pericardium group. No calcification was observed in the Biotube group.

**Figure 6:**
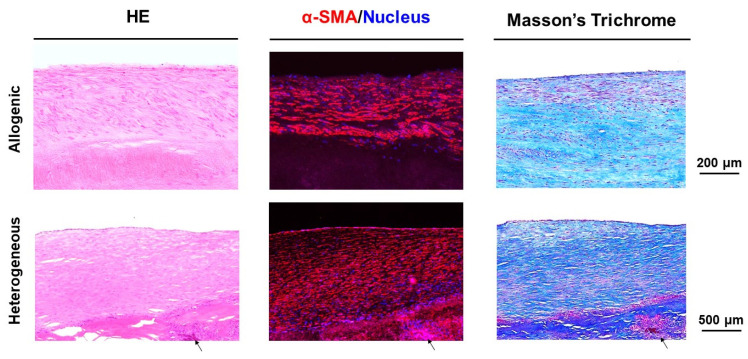
Histologic findings in the allogenic and heterogeneous groups. No immune cell infiltration in the allogenic Biotube group was observed. However, immune cell infiltration into the Biotube layer was observed in the heterogeneous Biotube group (arrows). Development of neoplastic cell layers positive for α-SMA was observed in both groups. Masson’s trichrome staining showed destruction of the Biotube layer due to inflammatory cells in the heterogeneous Biotube group.

Figure 6 shows the histologic findings of the allogenic and heterogeneous Biotubes. In the heterogeneous group, the histologic changes in most areas were similar to the allogenic Biotube findings described above. However, in the heterogeneous Biotube group, there was infiltration of inflammatory cells into the Biotube layer. Significant immune cell infiltration was observed in areas of macroscopic ulcer-like lesions. The development of the smooth muscle cell layer remains unaffected. However, Masson’s trichrome staining showed destruction of the Biotube layer due to inflammatory cells in the heterogeneous Biotube group.

## 4. Discussion

While the vascular prosthesis has excellent durability, retreatment due to infection is a problem in the long term. In general, reoperation is required in cases of graft infection but with a poor prognosis [1,2,8]. Aortic replacement with an aortic allograft can be effective but only in a limited number of cases due to issues with donation, as donated grafts are not always of sufficient quality and there are limited grafts available for surgery [2,9,10]. An ideal graft does not cause infection, immune reaction, or rejection. In addition, physical properties, such as ease of sewing, resistance to bending and rupture, resistance to aneurysm development and rupture, and resistance to high arterial pressure, are also required. Biotubes have the potential to serve as an alternative graft in cases of infection as they are autologous, tissue-engineered grafts that can be generated in vivo. In addition, the incidence of postoperative graft infection may be lower than with a vascular prosthesis; future research should focus on this issue.

The development of tissue-engineered vascular grafts has been reported from numerous institutions [3,11,12]. However, most are small-diameter grafts designed for peripheral vessels. In this study, we successfully performed ascending aortic replacement using Biotubes in the world’s first goat models. Biotube production requires no special equipment, materials, or facilities other than molds, and the process is simple. In addition, the Biotube diameter and wall thickness can be adjusted to create the required grafts by adjusting the mold size and gap. In peripheral vascular bypass models, to achieve flexibility, the mold was designed to obtain a graft thickness of approximately 0.8 mm [5]. In this aortic replacement model, the Biotube thickness was 1.5 mm because the graft must be durable enough to withstand aortic pressure. Optical coherence tomography was required to evaluate the Biosheet thickness because of the uneven thickness reported in a previous aortic valve study [4]. The thicker wall design stabilized the formation of the Biotube. In addition, the Biotube was permeated with 70% ethanol for dehydration before implantation. This process increases the collagen density of the Biotube. Previous studies have indicated that the Biotube becomes more durable than before treatment [13]. The Biotube designed in this study had a tensile strength of 22.18 ± 6.26 N. This is more than twice as strong as the previous Biotube used for peripheral vascular bypass [7]. In fact, the durability of this Biotube caused no failures during the 6-month observation period.

**Figure 7:**
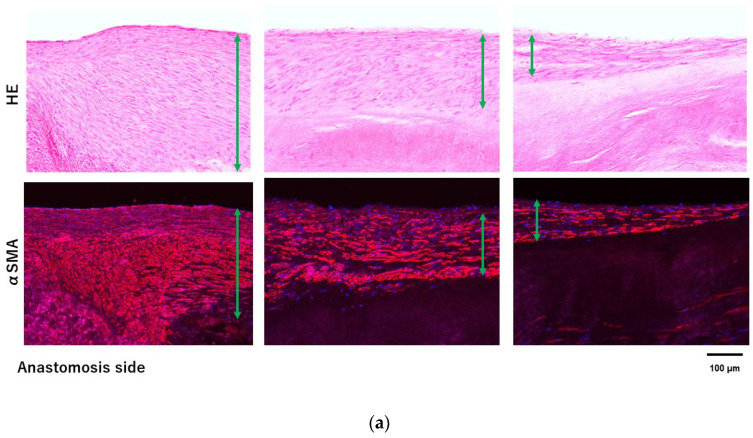

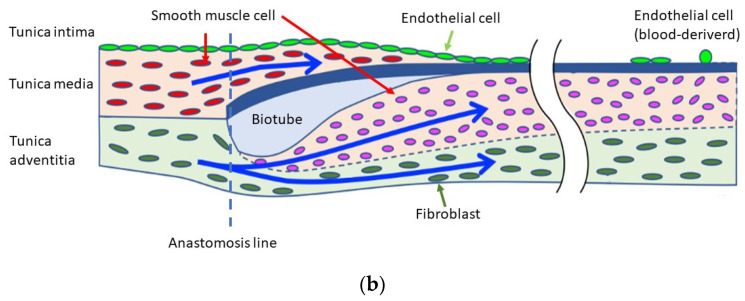
A neoplastic cell layer was observed along the Biotube surface in the allogenic Biotube group (**a**). There were more neoplastic cells around the anastomosis. This was also observed in the heterogeneous Biotube group. (**b**) Neoplastic cells, mainly smooth muscle cells, are expected to develop from the native aorta along the Biotube.

Collagen, the main component of Biotubes, is useful as a biomaterial for tissue-engineered grafts because of its low antigenicity and high biocompatibility [14,15,16,17]. The pericardium is a commonly used restorative material in clinical aortic surgery today [18,19]. In this study, we compared the Biotube with autologous pericardium. We observed neogenesis of endothelial cells, smooth muscle cells, and elastic fibers from the native aorta along the Biotube at 6 months. Neoplastic cells, mainly smooth muscle cells, developed more around the anastomosis of the native aorta and the Biotube. These findings suggested that smooth muscle cells and fibroblasts are regenerated on the Biotube scaffold from the transection of the native aorta (Figure 7). The regeneration process of the Biotubes was no less favorable than in the autologous pericardium group. Similarly, the development of these neoplastic cells was observed in aortic valve neocuspidization models [4]. In contrast, there were endothelial cells on the luminal surface, even in the areas where smooth muscle cells were poorly developed. As for the development of endothelial cells, it is suggested that they occur not only from transection of the native aorta but also from blood. Endothelization has been reported from other tissue-engineered grafts [20,21]. At 6 months, there was insufficient smooth muscle cell neogenesis far from the anastomosis. However, we expect that the regenerative process will continue beyond 6 months, forming the vascular structure, based on our previous aortic valve study.

In addition, no calcification was observed in the Biotubes. The autologous pericardium was calcified near the suture line as in the previous aortic valve model. Although the autologous pericardium had adequate durability, calcification remains one of the long-term problems. Glutaraldehyde, which is used as a pretreatment for pericardial reinforcement, is a well-known calcification factor [22,23,24]. The induction of regeneration without calcification is an excellent feature for a tissue-engineered graft. Thus, due to their favorable tissue affinity, Biotubes could be an effective scaffold for vascular regeneration.

Thrombus formation is one of the major problems in the development of vascular grafts [3,25,26]. Since Biotubes are formed only from collagen fibers, there are no endothelial cells on the surface immediately after implantation. There is also no heparin coating process, which is performed with vascular prostheses. These are unfavorable conditions for vascular grafts with respect to the prevention of thrombosis. However, as fibroblasts infiltrate through the pores, a high-density collagen layer forms on the Biotube luminal surface (core surface of the mold). The high-density collagen layer may have antithrombotic effects by decreasing blood component penetration [7]. However, we observed thrombus formation in the grafts with small-diameter Biotubes. Grafts for bypass are longer and, therefore, at risk of bending. We suspect that stagnant blood flow is a trigger for thrombus formation. Thus, we previously administered anticoagulants or antiplatelet agents to prevent early graft occlusion due to thrombus in the graft in our peripheral bypass study [5]. In contrast, there was no evidence of thrombus formation on the graft surface or of thrombo-embolization despite the nonuse of anticoagulation therapy in this aortic surgery. This may be due to inhibition of thrombus formation due to the high blood flow in the graft. In addition, the ascending aorta has a straight structure, less prone to turbulence and stagnation, which may decrease the potential damage to the intimal structures formed. If thrombus formation can be avoided in the following postoperative months during the development of endothelial cells, we believe that thrombus formation can be suppressed by endothelial cell function. Therefore, anticoagulation therapy is not considered necessary for straight aortic replacement.

The Biotubes were durable enough not to rupture under aortic pressure >6 months, as did the autologous pericardial rolls. In the heterogeneous Biotube group, although no rupture was observed during the observation period, there were numerous ulcer-like lesions on the lumen surface. A histologic evaluation showed infiltration of inflammatory cells in the area of ulceration. Possibly, the Biotube tissue was damaged due to an immune response to the heterogeneous collagenous tissue. In general, treatment with 70% ethanol should remove all cellular components [13]. However, the antigenicity was not completely eliminated, and the immune response was not suppressed in this model. Although we did not observe Biotube tearing or rupture due to tissue damage, it is possible that this damage could influence long-term Biotube durability. The duration of Biotube formation is disadvantageous in aortic surgery, as there are often urgent cases. In aortic surgery, iBTA technology would be more versatile if they could be transferred between species. For this purpose, further research into decellularization that does not affect the properties of the Biotube is required [27,28].

This study is limited by the small number of cases and the short observation period. Although no Biotube fracture was observed during the 6-month observation period, long-term durability (1 year) needs to be evaluated in the future. We are also interested in examining the changes in the physical properties of Biotubes and the progression of the regeneration process during long-term observation. In addition, the diameter of the graft produced by this mold is 12 mm, which is smaller compared with the diameter of an adult aorta. It is difficult to evaluate larger diameters to match the graft diameter to the goat aortic diameter. We considered that the current graft diameter would be difficult to use in adult aortic surgery. However, we believe it is appropriate for pediatric large vessel surgery. The development of large-diameter Biotubes is still in its early stages, and there is still plenty of room for improvement.

## 5. Conclusions

We successfully performed the world’s first ascending aortic replacement using a Biotube, an iBTA graft. Accordingly, Biotubes could be useful scaffolds for tissue regeneration in the aorta.

## Data Availability

The data are not publicly available due to their containing information that could compromise the privacy of research participants.
